# Bioelectrical Impedance Spectroscopy for Monitoring Mammalian Cells and Tissues under Different Frequency Domains: A Review

**DOI:** 10.1021/acsmeasuresciau.2c00033

**Published:** 2022-08-19

**Authors:** Sara Abasi, John R. Aggas, Guillermo G. Garayar-Leyva, Brandon K. Walther, Anthony Guiseppi-Elie

**Affiliations:** † Center for Bioelectronics, Biosensors and Biochips (C3B®), Department of Biomedical Engineering, Texas A&M University, 400 Bizzell Street, College Station, Texas 77843, United States; ‡ Cell Culture Media Services, Cytiva, 100 Results Way, Marlborough, Massachusetts 01752, United States; § Test Development, Roche Diagnostics, 9115 Hague Road, Indianapolis, Indiana 46256, United States; ∥ Department of Electrical and Computer Engineering, Texas A&M University, 400 Bizzell Street, College Station, Texas 77843, United States; ⊥ Department of Cardiovascular Sciences, Houston Methodist Institute for Academic Medicine and Houston Methodist Research Institute, 6670 Bertner Avenue, Houston, Texas 77030, United States; # ABTECH Scientific, Inc., Biotechnology Research Park, 800 East Leigh Street, Richmond, Virginia 23219, United States

**Keywords:** bioelectrical impedance, bioelectrical impedance spectroscopy, equivalent circuit models, tissue response, bioimpedance instrumentation, BIA/BIS wearables, Internet of Medical Things, clinical bioimpedance

## Abstract

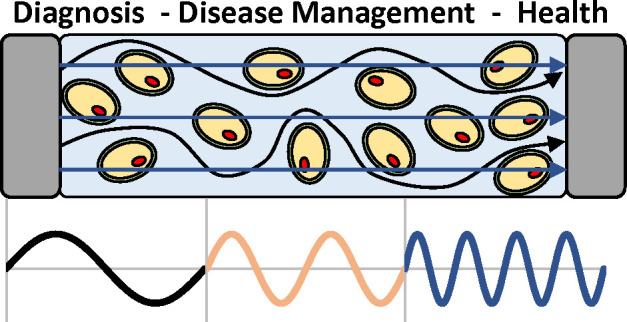

Bioelectrical impedance analysis and bioelectrical impedance spectroscopy (BIA/BIS) of tissues reveal important information on molecular composition and physical structure that is useful in diagnostics and prognostics. The heterogeneity in structural elements of cells, tissues, organs, and the whole human body, the variability in molecular composition arising from the dynamics of biochemical reactions, and the contributions of inherently electroresponsive components, such as ions, proteins, and polarized membranes, have rendered bioimpedance challenging to interpret but also a powerful evaluation and monitoring technique in biomedicine. BIA/BIS has thus become the basis for a wide range of diagnostic and monitoring systems such as plethysmography and tomography. The use of BIA/BIS arises from (i) being a noninvasive and safe measurement modality, (ii) its ease of miniaturization, and (iii) multiple technological formats for its biomedical implementation. Considering the dependency of the absolute and relative values of impedance on frequency, and the uniqueness of the origins of the α-, β-, δ-, and γ-dispersions, this targeted review discusses biological events and underlying principles that are employed to analyze the impedance data based on the frequency range. The emergence of BIA/BIS in wearable devices and its relevance to the Internet of Medical Things (IoMT) are introduced and discussed.

## 1 Introduction

Bioelectrical impedance analysis (BIA), bioelectrical impedance spectroscopy (BIS), and bioimpedance tomography (BIT) have firmly established themselves as appropriate modalities for measurement and monitoring to accommodate diagnostics and prognostics in clinical care. With established applications in body composition analysis, where the goal is to aid diagnosis, monitor disease progression, support therapeutic interventions,^1^ and better elucidate disease mechanisms,^2^ bioimpedance is now being fervently applied at the cellular,^3−6^ tissue,^7−9^ and organ levels^10,11^ with similar goals in mind. While acknowledged within, this review is not concerned with whole body composition analysis to which the reader is directed to excellent recent reviews on this topic.^12,13^ Of emerging significance is bioimpedance monitoring with simultaneous therapeutic intervention,^10^ perioperative and postoperative monitoring,^14,15^ and surgical guidance,^16^ which is the focus of the present review.

While possessing many advantages, BIA is nonetheless plagued with some limitations. Among these are the fact that all materials possess the property of impedance and that the impedance response of “tissue under test” (TUT) is not biomolecularly, compositionally, or physicochemically specific. Accordingly, there is need for sophisticated ratioing and/or referencing techniques intended to reveal changes in BIA and similarly sophisticated mathematical techniques and models to allow quantitative interpretation of acquired data. Being a technique that employs radio waves, bioimpedance requires contacting electrodes and hence the temporal quality of the electrode–tissue interface, and the associated contact impedances present a measurement challenge. The finite size and density of contacting electrodes influences spatial resolution and time between updates when considering continual measurements. Many of the foregoing are areas of active fundamental research, engineering development and optimization, and preclinical and clinical study.

According to the PubMed [Search query: “bioimpedance” OR “bioelectrical impedance” AND “clinical” (as of July 23, 2022)], the number of published articles that discuss bioimpedance grown exponentially (*R*^2^ = 0.955): 150 in 2000, 207 in 2005, 241 in 2010, 470 in 2015, and 761 in 2020. This growth is associated with the emergence of the Internet of Medial Things (IoMT) and wearable devices and in a robust movement toward clinical applications. Many clinically oriented applications relate to monitoring pathophysiological events such as plethysmography, the measurement of limb blood flow under stimulated relative to baselined conditions, and encephalography, the examination of the brain by impedance following the withdrawal of cerebrospinal fluid and introduction of air or an inert gas.

Fundamental challenges in BIA are centered around the underlying sources and mechanisms of resistance and reactance in resolving pathophysiology from physiological dynamics. Unlike solid state or soft-condensed biomimetic materials, real living tissues are dynamic with multiple competing/complementary biochemical pathways and molecular events that seek to maintain homeostasis. Real tissues are generally inhomogeneous and are not isotropic and therefore subject to spatial variations. The size of physically contacting electrodes therefore serves to establish the volume element or voxel of the TUT and thus influences the sample resolution. Accordingly, the temporally evolving observed dielectric dispersions are not readily traceable to a particular structural component or condition. In principle, bioimpedance analysis should permit quantitative assessment of tissues based on the intrinsic electrical properties of tissue components (fat, muscle, bone, extracellular fluid, polarizable molecules, ion concentrations, etc.) and may thus be discriminating with regard to the pathophysiology at the molecular, cellular, tissue and organ level according to the applied frequency range. The very principle also defines the problems. Tissue composition is not a priori known and varies among patients. Also, molecular/cellular compositions are variable according to the pathophysiology and physiological dynamics.

Nonetheless, several practical applications have emerged in recent years. These include laboratory-based bioelectronic diagnostic techniques to address cellular growth and proliferation under a plurality of cell culture conditions^17−19^ and bioimpedance spectroscopy of small biopsied tissues promise to identify tissue atypia.^20,21^ In addition to mammalian tissue, bioimpedance has been utilized to study the properties of plants such as hydration,^22^ nutrient transport,^23^ and ripeness.^24^ For a detailed review on bioimpedance of plants, the reader is directed to previous references.^25−27^

The present timely targeted review takes a critical view of bioimpedance largely from the perspective of the underlying biomolecular and biophysical response mechanism and focuses exclusively on how an appreciation of these response mechanisms enables BIA/BIS to be an effective tool for the monitoring of cells in culture and native tissues and organs. This is not intended as a review of electrochemical or electrical impedance spectroscopy (EIS). This targeted review of the use of EIS for the measurement and monitoring of native tissues introduces EIS basics to allow an appreciation and interpretation of tissue specific responses to the unique frequency domains used in interrogation.

Bioimpedance basics have recently been thoroughly reviewed,^28,29^ is the subject of textbooks^30^ and monographs^31^^32^ and so is limited in its presentation here. Following a short, general survey of these basics, the impedance characteristics of biological tissues are surveyed and rationalized. The interrogating frequency (impedimetry) (BIA) and/or frequency range (impedance spectroscopy) (BIS) determines the response of the tissue under test (TUT). Accordingly, this review takes the unique perspective to present BIA/BIS/BIT protocols/techniques, phenomena, instrumentation, and equivalent circuit modeling in terms of the interrogating frequency range and the corresponding response according to the alpha (α ∼ 10 Hz to 10 kHz) and beta (β ∼ 10 kHz to 10 MHz) ranges, the delta range (δ ∼ 100 MHz to 10 GHz), and the gamma range (γ > 10 GHz). Additionally, this review presents a survey of four clinically significant applications, namely, edema, pulmonary capacity, cardiovascular monitoring, and malignancy diagnostics, that serve to contextualize and illustrate the forgoing concepts. Finally, an analysis of current wearable bioimpedance technologies is presented, along with how they fit into the emerging IoMT.

## 2 Background

Impedance is the property of a device, material, or tissue reflective of its ability to resist the movement of charge, ionic or electronic, in response to an interrogating, alternating electric (AC) field. In general, electrical impedance (EI) is a four-electrode measurement sometimes employing two-electrodes performed using a lock-in amplifier and function generator or frequency response analyzer (FRA), the working or injection electrode (WE) and the counter or sink electrode (CE). Electrochemical impedance (ECI) is distinguished in its requirement for a third or reference electrode (RE) and additionally employs a potentiostat. The RE contributes a known and stable half-cell potential to which the potentiostat references its interrogating voltage. Two-electrode measurements, when used self-consistently, provide for accurate and reproducible relative measures of impedance, however, being nonreferenced the absolute values of impedance may vary from system to system or from TUT to TUT, as the open circuit potential at which the two-electrode measurements are made may itself vary.^33,34^

**Figure 1:**
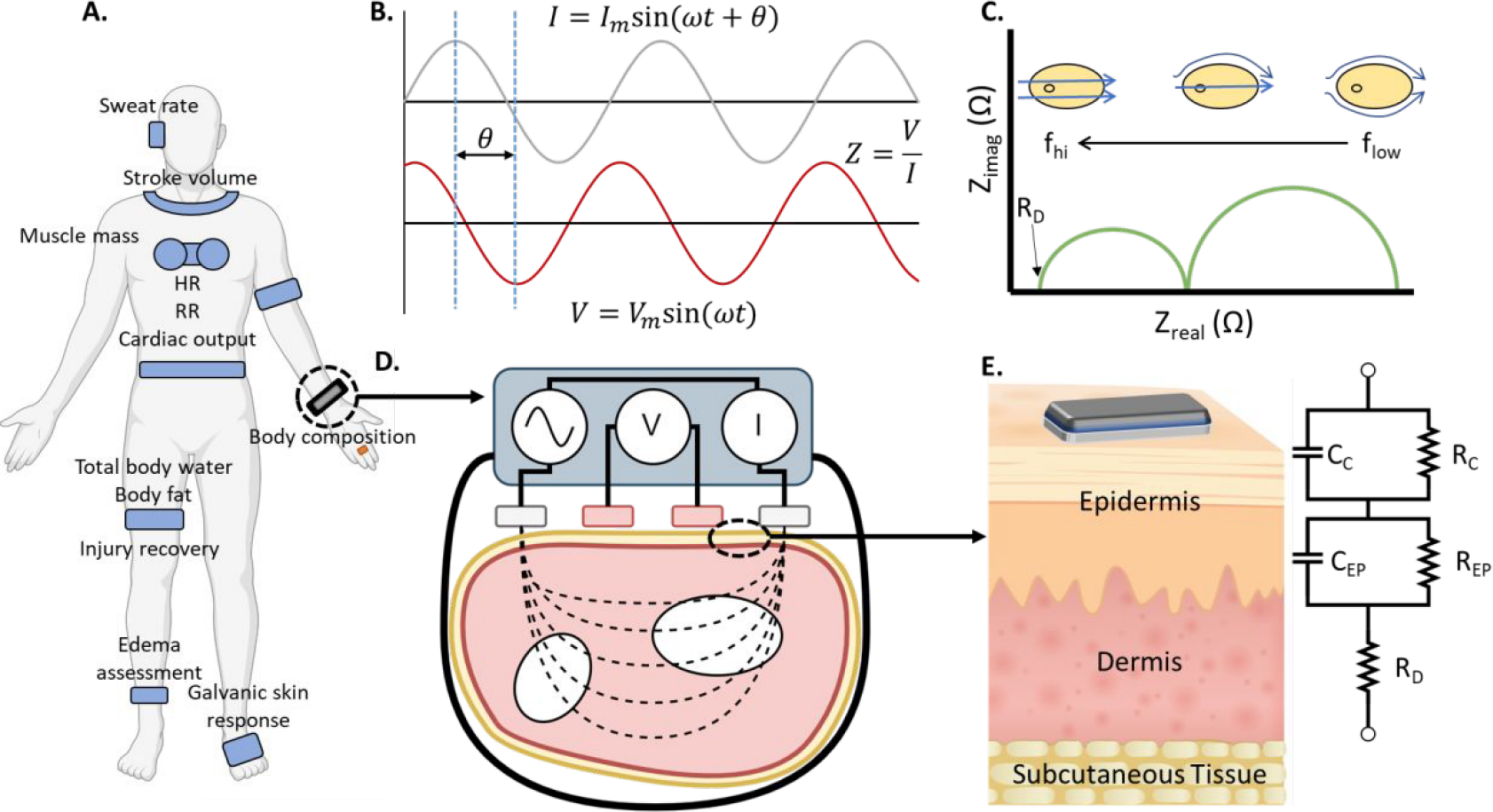
(A) BIA/BIS has found use in a number of wearable devices, which have been designed to interrogate and monitor various parts of the body, yielding health parameters for both clinical and nonclinical applications. Adapted with permission under a Creative Commons CC License from ref (37). Copyright 2019 Frontiers Media. (B) Fundamental approach to BIA/BIS measurements uses current injection via low amplitude sine wave (at single (BIA) or multiple frequencies (BIS)) and the measurement of resultant voltage to yield the transfer function, impedance (*Z*). (C) Resultant impedance spectrum measured across a range of frequencies with two dispersions is common when measuring BIS as *Z* within tissues changes as a function of interrogation frequency. (D) Implementation of a four-electrode (tetrapolar) wrist-worn bioimpedance measuring device wherein outer current injection electrodes are used to create a signal, and voltage sensing inner electrodes monitor the resultant signal. Four-electrode systems are considered superior to their two-electrode counterparts for their ability to reduce contact resistance measurement error. (E) Equivalent circuit analysis is used to model and extract physiologically or compositionally relevant information from measured bioimpedance data. For a wrist-worn device, impedances such as contact resistance and contact capacitance must be considered, as well as resistances and capacitances associated with the epidermis (*R*_EP_, *C*_EP_) and dermis (*R*_D_) layers.

Figure 1 schematically illustrates a typical setup for the measurement of BIA and BIS. The instrumentation delivers a varying sinusoid and interrogating potential of specified frequency at the CE:
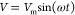
1At the WE, the ensuing AC response is measured:
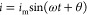
2Here, ω is the radial frequency, *V*_m_ is the maximum voltage at the peak, and *V* is voltage at any given instant. The AC response (*i*) is characterized by both its amplitude (*i*_m_) and its phase shift (θ) with respect to the applied AC voltage. The transfer function, the ratio *V*/*I* in the complex plane, measures the impedance (*Z*). The ratio of the amplitudes of the applied and the response signal (*V*_m_/*i*_m_) and the phase shift between these signals (θ) is used to determine the impedance of the TUT. The real component of impedance is the resistance, *R*, and the imaginary component, *X*, is the reactance. The addition of which results in the impedance:
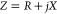
3When measured over a range of frequencies, this produces an impedance spectrum. Alternatively, the impedance can be presented as
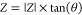
4where |*Z*| (magnitude) equals
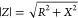
5and the θ (phase) equals
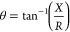
6It is commonplace to interpret impedance data based on defining an appropriate equivalent circuit that best fits the acquired frequency-dependent real and imaginary data. An equivalent electrical circuit consists of a specific arrangement of resistors, capacitors, and inductors in serial, parallel or combinations thereof with a characteristic time constant. The reactance, *X*, describes the frequency dependent part of the TUT that behaves as a capacitor or inductor while the resistance, *R*, describes that frequency independent part of the TUT that behaves as a resistor (Figure 1E).

The TUT can manifest impedance data reflective of several time constants, but what is revealed is dependent upon the frequency range used for interrogation. To be useful, the elements in an equivalent circuit should always have physicochemical significance inherent in the properties of the TUT. For example, a pair of noble metal electrodes in contact with a physiologically relevant electrolyte solution (e.g., oxygenated phosphate-buffered saline 7.4) may be represented by an equivalent circuit for which a resistor represents faradaic charge transfer across the electrified interface (*R*_CT_), a parallel capacitor represents the Helmholtz electric double-layer (*C*_DL_) and a series resistor represents the solution resistance (*R*_SOL_). Considering such a simple, illustrative system, the total complex impedance (*Z*_tot_), the real impedance (*Z*′) and the imaginary impedance (*Z*″) are a function of applied frequency (ω = 2π*f*) and is mathematically represented by the following (eqs 7, 8, and 9):^35,36^
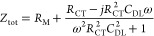
7
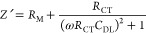
8
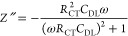
9

**Figure 2:**
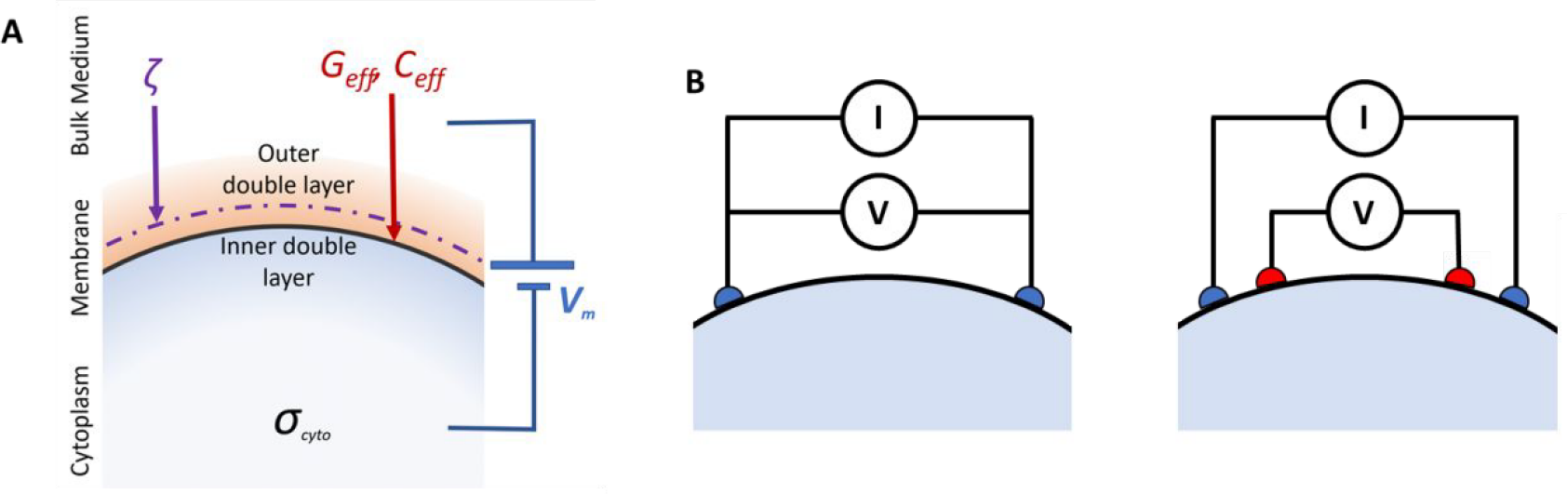
(A) Schematic illustration of the origins of the transmembrane impedance in response to an interrogating voltage, *V*, showing the leaky dielectric membrane, the inner and outer Helmholtz double layers and the Gouy–Chapman layer. (B) Two-electrode and four-electrode setups for bioimpedance measurements.

The major challenges in defining the frequency range of interest in BIS techniques are (i) contact impedances—physical contacting electrodes are required which introduces contact impedances, often confounding the impedance of the TUT and so necessitates judicious choice of electrode materials, (ii) electrified interfaces—the electrified electrode-tissue interface with its structured inner and outer Helmholtz plane and diffuse Gouy–Chapman layer establishes its own equivalent circuit contribution that must be satisfactorily studied and modeled or nullified to allow a response of the TUT to be extracted (Figure 2), (iii) tissue inhomogeneity—real tissues are not homogeneous and isotropic, hence are subject to variations in all three axes, (iv) sample volume—the volume element or voxel that is sampled by the contacting electrodes serve to define the sample resolution, (v) event time scale—the time scale of events that changes ion concentration, ion mobility, or abundance of polarizable molecules within the tissue relative to the time scale of an impedance measurement serves to establish the needed update frequency of measurements, and (vi) nonspecificity—impedimetric measurements are not molecularly or feature specific with changes arising from a plurality of possible sources. The forgoing challenges require the use of proper experimental referencing conditions and controls to establish reproducibility and the use of mathematical constructs to extract correlative or causative data.

## 3 BIA/BIS Techniques

### 3.1 Interrogation Protocols

**Figure 3:**
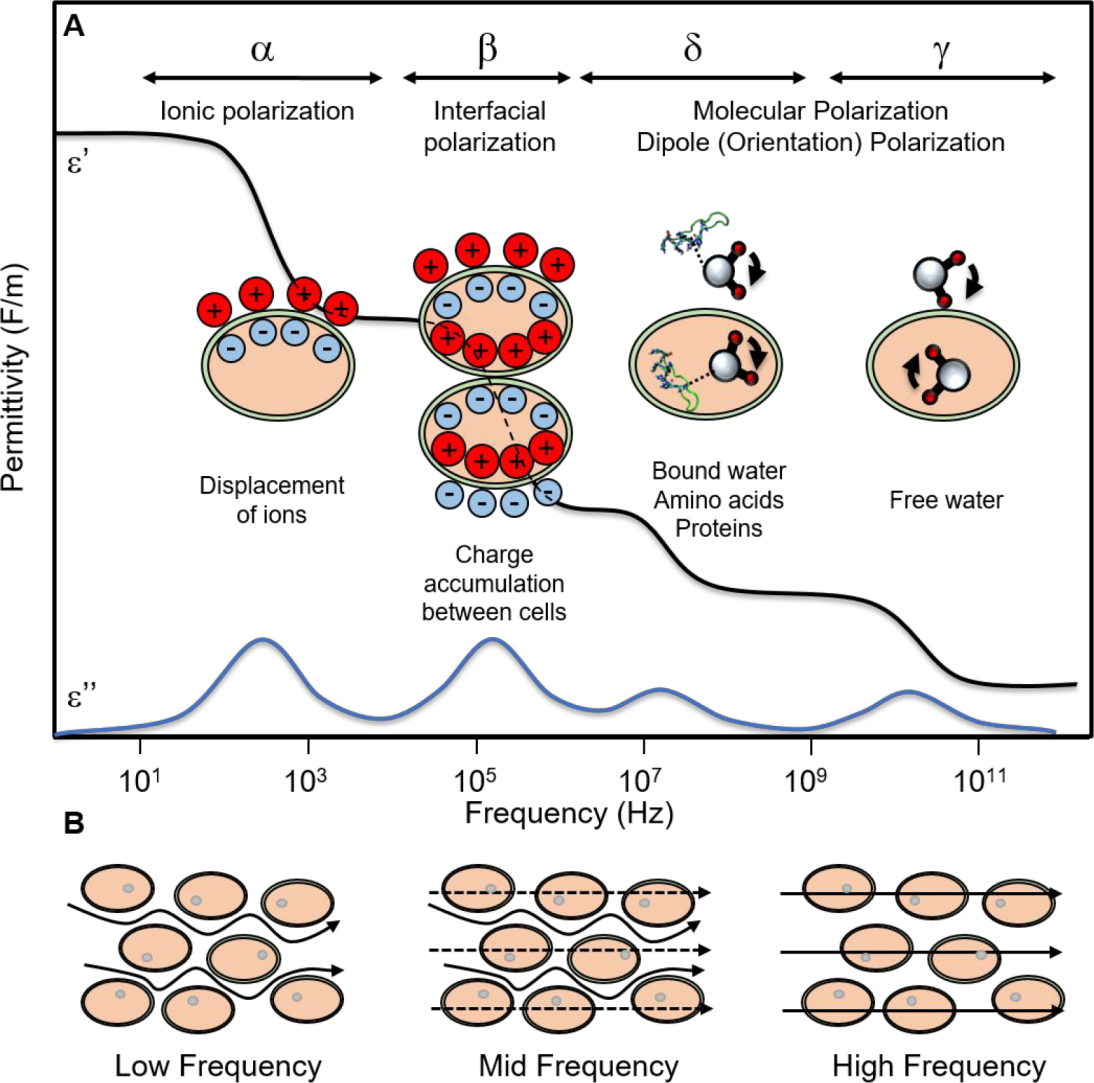
(A) Real and imaginary components of dielectric permittivity of biological tissue as a function of frequency (ε = ε′ + *j*ε″). Dispersions of the permittivity occur in four major frequency windows (α, β, δ, γ). At low frequencies (α region), the dispersion is dependent on displacement of the variety of solvated ions due to ionic polarization. In the β region, the dispersion is dependent on charge accumulation between cells due to interfacial polarization. In the δ region, the dispersion is dependent on the behavior bound water and amino acids to molecular and dipole polarization. In the γ region, the dispersion is dependent on free water due to dipole polarization. (B) Path of current through cells is dependent on applied frequency. At low frequencies, current travels around cells in the intracellular space. At middle frequencies, current continues to pass around cells, but begins to act on the space within the cell membrane. At high frequencies, current affects the space inside the cells entirely.

One area of concern when applying voltage/current to biological tissue, even the small values associated with BIA/BIS, is its unintentional effect on the tissue. There is abundant literature available which discusses the limits of magnitude/frequency that may be applied by a medical device.^38,39^ This limit varies based on the frequency of measuring voltage/current, the type of tissue, electrode material and configuration, etc. According to the International Electrochemical Commission (IEC), the maximum leakage current allowed for a medical device is 100 μA at working frequencies of 0.1 Hz to 1 kHz. In general, the threshold of allowed current is higher at extremely low and high frequencies because of the time given to the tissue to accommodate and dissipate the effect of EF in the former and the capacitive properties of membranes which result in a finite response time at the latter range.^40^ However, the maximum allowed current may exceed recommendations during therapeutic/diagnostic applications such as bioimpedance tomography (BIT), although most human trials stay at or below that limit to ensure safety. In the following sections we associate tissue responses with the frequency ranges of interrogation.^41^Figure 3 shows an overview of the dominant electrical phenomena associated with the tissue response (in terms electrical permittivity) that correspond to the four dispersions: α, β, δ, and γ.

#### 3.1.1 Alpha (∼10 Hz to 10 kHz) and Beta (∼10 kHz to 10 MHz) Ranges

Tissue is more susceptible to destruction when utilizing low-frequency electrical current in the α and β ranges. The risk of ventricular fibrillation is highest for frequencies from 10 to 200 Hz. While the risk is slightly reduced at 1000 Hz, it rapidly decreases at frequencies above 1000 Hz.^42^ At zero frequency (DC), the electric field (EF) cannot be coupled into tissue in the absence of a physical contact between the electrode and tissue. Using AC, the sensation threshold is not linear with respect to frequency. Several mechanisms through which EF applies its effect on biological tissues have been suggested and elaborated, such as the torque force applied to charged molecules in an EF and dielectrophoretic forces.^43^ The application of EFs at low frequencies generates heat in biological tissue via energy absorption in the cells which would be considered as a limit to the magnitude of electric signal because biochemical reactions are sensitive to small temperature changes with a temperature coefficient on the order of 3% K^–1^.^40^ The electrical signal that is applied to measure bioimpedance must not activate or up-regulate biochemical processes within the cell as it could result in a disturbing response that convolutes the measurement. This is of utmost importance when electrically excitable cells such as neurons are involved as these cells have different thresholds of activation.^44^ An in vitro study indicates a significant response in mammalian hippocampus by an EF of around 1–5 V/m.^45^

Another consideration in this frequency range is measurement time: as the interrogation frequency gets lower, the time required for impedance data collection increases. For example, a single interrogation period of a 0.1 Hz wave is 10 s (*T* = 1/*f*). Therefore, if the TUT under examination changes at a rate faster than the sampling frequency, the collected data will be nonexplanatory. The longer sampling time should not be at the expense of losing critical temporal data. Therefore, if bioimpedance is intended for real-time monitoring, the data acquisition time at low frequencies must be duly considered when designing the interrogating frequency range.

#### 3.1.2 Delta (∼100 MHz to 10 GHz) and Gamma (>10 GHz) Ranges

Bioimpedance measurements in the δ and γ ranges are far less common due to the requirement of complex instrumentation and the fact that most of the physiological processes of interest are measured in the α and β ranges. Nevertheless, measurements regarding free/bound water in cells (hydration status) often utilize the δ and γ windows for characterization. Hussein et al. found that breast cancer tissue showed distinct bioimpedance signatures when measuring over the range of 200 MHz to 13.6 GHz.^46^ A study by Lazebnik et al. on the characterization of breast tissue yielded important information on proper interrogation of tissues at high frequencies.^47^ A coaxial probe (OD = 3 mm) was placed in direct contact with tissue for measurements over 0.5–20 GHz. In fact, measurement of the dielectric constant at 5 GHz gave the ability to discern percentages of tissue composition (adipose, glandular, fibroconnective).^47^ When coaxial probes are used for BI measurements, the depth of insertion into tissue and diameter of the probe become important factors (due to field fringing effects) alongside the measured frequency. For probes with diameter of 3.58 mm, the sample must be >3.0 mm thick, and for probes with diameter of 2.2 mm, the sample must be >1.5 mm thick.^48^

High-frequency measurements have also been used for in vitro characterization of cell culture systems. Schmid et al. created a custom bioimpedance spectroscopy system to measure response of hanging drops of microtissue spheroids.^49^ The system, which used 100 Hz to 40 MHz, covered frequencies far beyond the β range but slightly short of the δ range. However, their experiments into the size of electrodes used at high frequencies give insight into the sorts of interrogation protocols required for probing in δ and γ ranges. In particular, the volume of the drops (μL) was discernible by using electrodes that were spaced 1.8 mm apart at very high frequencies (>10^7^ Hz). Electrodes that were spaced 0.8 mm apart were able to discern changes in volume at 10^4^ – 10^6^ Hz). Similar work by Bagnaninchi combined high-frequency impedance spectroscopy (20 MHz to 1 GHz) and Fourier domain optical coherence tomography (FDOCT) to monitor cell volume fractions and dielectric properties of human adipose derived stem cells (hADSCs) using an open-ended coaxial probe.^50^ In their system, the coaxial probe (OD = 3.5 mm) was much smaller than the testing platform in order to mitigate fringing effects of fields at the very high frequencies. The results showed a correlation between the number of cells in the system and the measured complex permittivity.

### 3.2 Biological Phenomena

#### 3.2.1 Alpha (∼10 Hz to 10 kHz) and Beta (∼10 kHz to 10 MHz) Ranges

Alpha (α) and beta (β) ranges are regarded as the low-frequency regime for bioimpedance. Most literature use these frequency ranges to study biological processes because the impedance in this range reflects important information on the structure and composition of tissue per se as well as its time-dependent relaxation behavior. Vesicles, proteins, and any charged entity in general resemble a colloidal particle suspended in an electrolyte solution (intra/extracellular fluids). These charged colloidal particles are surrounded by counterions which are controlled by the gradient of the electrolyte and electrical potential of the surface. Electrochemically, the counterion layer behaves like a double layer capacitance leading into a dielectric dispersion at low frequencies because of the diffusion-controlled relaxation regarded as α-dispersion shown in Figure 3. Under the influence of a built-in electric field, ions in this layer are strongly bound with limited mobility. The application of an external electric field exerts a force on polar biomolecules, causing them to reorient while their movements are restricted by interfaces inside the material, resulting in a dielectric relaxation (α-dispersion).^51^ Counterion layers that establish a built-in electric field also form around and within the lumen of pores in the cell membrane. In the presence of an external field perpendicular to the built-in field and when the pore size is larger than the counterion hydrodynamic thickness, ions accumulated adjacent to the pore can move through membrane pores, with more movement of ions producing stronger dispersion.^52,53^Table 1 provides a summary of various biological components and the corresponding frequency range over which their interrogation elicits a measurable response.

**Table 1:** Relevant Dispersion Ranges for Various Biological Molecules^a^

		dispersion range			
contributing biomaterial element		α	β	δ	γ
water and electrolytes					•
biological macromolecules	amino acids		•	•	•
	proteins		•	•	•
	nucleic acids	•	•	•	•
vesicles	surface-charged	•	•		
	nonsurface-charged		•		
cells with membrane	+fluids free of protein		•		
	+tubular system	•	•		
	+surface charge	•	•		
	+membrane relaxation	•	•		
	+organelles		•	•	•
	+protein		•	•	•

a Reproduced with permission from ref (54). Copyright 2012 AAPM.

The presence of nonhomogeneous components and ionic activities within the tissue determine the electrical behavior of tissue at frequencies in the β range. This dispersion arises from interfacial depolarization of cellular membranes above 100 kHz and is described by Maxwell–Wagner mechanism which arises from accumulation of charges and restriction of their movement at the interface of materials with different electrical properties (Figure 2). In an external electric field, movement of ions in tissues is inhibited by various barriers and layers, such as the tight junctions of endothelial or epithelial layers or cell membrane, which result in a capacitive charging effect that reflects as a dispersion in the β range. The level of charge accumulation, hence dispersion, depends on structure and composition of the tissue. This results in each type of tissue having a distinctive frequency-dependent electrical characteristic summarized in Table 2. The response of tissue in the β range has long been modeled as spherical particles suspended in a dilute electrolyte and as multilayered materials.^55^ The former is appropriate for describing the electrical behavior of tissues such as blood or cells in suspended culture, while the latter is ascribed to tissue with tightly packed layers of cells such as skin or muscle. The β relaxation frequency is largely impacted by the ratio of thickness of insulating layer, e.g., cell membrane, to the electrolyte layer, e.g., cytoplasm; as this ratio decreases, the dispersion shifts to lower frequencies and becomes stronger. The thickness of the electrolyte layer could be related to the water content of tissue, hence as tissue disintegrates during necrosis or aging; its β-dispersion is hugely reduced because of water loss. Similarly, as tissue swells, such as in trauma induced edema, its β-dispersion is increased because of water retention. All tissues have β-dispersion which are qualitatively similar. Higher lipid content of tissue and/or lower electrolyte content smoothens the impedance changes with frequency. The β range is the most widely used range in bioimpedance studies of biological tissues since it does not have the technical complication of low-frequency (α) measurement yet provides in-depth information about the tissue and its structure.

**Table 2:** Bioimpedance Characteristics of Different Tissue Types (Measurements Taken Ex Vivo)

	resistivity (Ω·m)										
tissue	0 Hz	10 Hz	50 Hz	10 kHz	50 kHz	100 kHz	500 kHz	1 MHz	10 MHz	1 GHz	source
brain											(56,57)
gray matter	15	13.5	13.3	10	7.7	6.9	6.7	4.8	2.9	1.1	
white matter	16.7	20	18.9	15	12.8	10.3	10.3	7.1	4.8	1.7	
fat	50	40	40	40	40	40	40	40	40	20	(58,59)
muscle											(60,61)
cardiac	10	10	5	2.2	2.1	2.1	2	1.8	1.7	1.1	
skeletal	2.5	4.4	3	2.9	2.9	1.3	2.2	1.1	1.1	1.1	
liver	14.3	8.3	8.3	7.7	6.3	5.6	4	3.3	2.1	1.2	(56,59)
lung	7.1	33.3	25	10	10	10	6.7	6.7	3.3	2.2	(57)
kidney	10	10	6.7	4	3.5	2.9	2.5	2.2	1.3	0.7	(57,60)
spleen	10	20	14.3	4.2	3.2	1.6	1.6	1.6	1.2	0.8	(60)
skin											(56,58)
dry		5 × 10^4^	3 × 10^4^	1 × 10^4^	5 × 10^3^	50	20	2	1.4	1.2	
wet	0.2	0.2	0.2	0.2	0.2	0.2	0.2	0.2	1		
bone	16.7	14.3	12.5	12.5	12.5	12	11.5	11	11	6.9	(61)
blood	1.6	1.5	1.4	1.4	1.4	1.6	1.3	1.4	0.9	0.7	(62)

The use of impedance to study ischemia in dissected and perfused liver and heart has offered new insights into the low-frequency behavior of the unique properties of these biological tissues and is illustrative of impedimetric monitoring of changing tissue dynamics. In heart tissue where muscle fibers (cardiomyocytes) do not have tight junctions, impedance measured at 100 Hz (α region) did not considerably change during the first 200 min upon onset of ischemia. After about 300 min post-ischemia, there was a sharp increase in impedance to about 2-fold due to possible swelling of cells and narrowing of the extracellular space.^63^ Similarly, ischemia was also studied according to the impedance in the β range. In the heart (muscular tissue), because of ischemia, the β-dispersion initially observed at 100 kHz shifted to 3 kHz. It should be noted that although the 3 kHz dispersion falls within the α region, the underlying reason for the appearance of this dispersion is the structural changes in the tissue following ischemia hence the dispersion is still a β-dispersion. Changes in the membrane structure disturb biomolecule and ion distribution in inter- and intracellular spaces causing the dispersion to occur at a lower frequency. The 10 MHz impedance measured in the β range of heart tissue experiencing ischemia showed a steady but relatively smooth decrease during the first 300 min followed by a plateau indicative of organ death as the ATP reserve became exhausted, and the accumulation of metabolic products served to reduce the tissue impedance.^63^

In liver tissue, where hepatocytes are connected closely via tight junctions, the impedance spectra indicated three dispersions: at 25 kHz (β-dispersion), 7 Hz (α-dispersion), and 0.1 Hz (sub-α-dispersion). The sub α-dispersion at 0.1 Hz was believed to reflect conducting pathways in the extracellular space and quickly disappeared as cells narrowed these pathways. This dispersion was only observed during a short period of time (∼20 min) post-ischemia. The impedance corresponding to the α-dispersion gradually increased during the progression of ischemia and the α-dispersion finally disappeared. The α-dispersion at 7 Hz disappeared after 200 min post-ischemia. At this frequency, the current mostly existed through tight junctions and the extracellular space. With the progress of ischemia, swelling of cells closed tight junctions followed by necrosis with accompanying disintegration of the cell membrane caused a change in tissue acidosis and an increased availability of ions within the tissue. Studying the time course of ischemia at a frequency between the α- and β-dispersions (193 Hz) provided additional information. At this frequency, the current was established through both the intra- and intercellular pathways. During early onset ischemia, cells use ATP reserves to maintain membrane function and hence the impedance remains constant. Impedances at 7 and 193 Hz both plateaued after 150 min, when ATP reserves were exhausted and membrane function could no longer be maintained. This is the hallmark of membrane destruction and cell death, beyond which the tissue cannot be revived. In addition, the shift of the β-dispersion (∼ 20 kHz) to lower frequencies was still observed (like that of heart tissue) but at a much lower strength compared to heart tissue. Unlike the α-dispersion which disappeared post-ischemia when the tissue was dead, the β-dispersion became stronger as the ischemia progressed.^63^

Studies that use bioimpedance spectroscopy to monitor dissected biological tissue, e.g., to infer or establish meat freshness, show the presence of the α-dispersion if the measurement frequency is low enough. Following dissection, the real part of the impedance (resistance of tissue) increases over a 300 min period as the tissue goes into rigor. Beyond this time, necrosis occurs, and the resistance continuously decreases accompanied with weakening of the α-dispersion. The weakening is due to an osmolarity increase in the extracellular space because of ions and biomolecules released from cells into the extracellular space while the water content of the tissue is reduced.^64^ The low-frequency resistance of a dissected tissue increases initially due to edema, the result of the accumulation of fluids within the tissue bed; the effect of edema then surpasses the baseline intercellular ionic contributions as the membrane loses its control on ion movement resulting in a decrease in the trans-membrane resistance; eventually as cells lyse and cell membranes rupture, the resistance again drops. This chain of events causes the disappearance of the α-dispersion post-mortem.^64^

Impedance measurements of live human patients similarly show α- and β-dispersions. Despite the anisotropic nature of impedance, the dispersion frequencies are not appreciably affected.^65^ The contribution of cellular morphology and arrangement in the bioimpedance are indicative of the potential of this metric to distinguish between tissue types. Studying a rat model, Dean et al. reported an α-dispersion around 100 Hz for the lung and 10 Hz for mesenteric vessels.^8^ In the low-frequency regime, strong anisotropic properties of muscle tissue render the impedance dependent on the direction of applied electric signal (hence electrode placement). The BI measured across or along the muscle fiber, transverse vs longitudinal, are different by a factor of π/2, since the low-frequency current travels a longer distance circumventing the fiber in transverse mode. Such differentiation in the directional impedance weakens with aging (3–4 days post-mortem) as the electrical barrier of cell membranes disappears allowing the current to choose a shorter path through the cell.^66^ Such a difference is observed principally in the α-range, as the current corresponding to low frequencies is preferentially established through the intercellular space which differs in path length based on the arrangement of the electrodes. In excised muscle, the β-dispersion disappears 3 days post-excision due to disintegration of the tissue.

#### 3.2.2 Delta (∼100 MHz to 10 GHz) and Gamma (>10 GHz) Ranges

The main consideration at high-frequency BIA/BIS measurements is the behavior of water (free and bound)^67^ as it has a dispersion of polar origin at ∼20 GHz. Seventy percent of body weight is made of water, and it plays a determinative role in electrical properties of tissues, biological solutions, and proteins at ultrahigh frequencies: the δ and γ ranges. Water content is a helpful marker for evaluating the health of cells, as it is known that some underlying conditions, such as cancer, particularly solid tumors, causes a change in water content of the tissue.^68^ In case of cancer and malignancy, the correlation of the defect with bioimpedance is not yet fully understood, changes in water content (perceptible at high frequency) along with cell–cell interactions and extracellular matrix reshaping (perceptible at lower frequencies) urge both caution and promise in using diagnostic bioimpedance to identify, grade, or stratify solid tumors. Early research studying electrical properties of malignant and normal tissues of different types confirm the potential of using bioimpedance for detection of malignancy. Results suggest that the efficiency of this technique is different based on the tissue structure; a bioimpedance scan in the δ regime, 50–900 MHz, showed the highest discrimination between malignant and normal tissues in mammary and lowest in kidney tissue.^69^ Such changes are further confounded with the composition of tissue, e.g., lipid content.^47^ The bioimpedance measured over δ and γ frequency range could be efficiently used to monitor events involving blood. Due to the high water content of blood, a reduction in the blood volume results in the weakening of γ-dispersion magnitude.^70^ Electrical properties of various tissues with high water and blood content are quite similar in this frequency regime with the permittivity and conductivity of the tissue changing only slightly between frequencies of 100 MHz to 10 GHz. Above this frequency (>10 GHz), the polar properties of water and its γ-dispersion at ∼20 GHz come into play and result in a rise of the tissue conductance.

Unlike α and β ranges, where the cell membrane serves as a barrier to free ion transport and dominates the electrical response, for δ and γ frequency ranges, the cell membrane is shear, as the membrane capacitance becomes negligible at these higher frequencies. The capability of microwave range electrical signals to pass beyond the cell membrane has allowed probing of cytoplasm status and intracellular events. A crucial application is in the food industry where BIS over the range of 0.5–20 GHz is used to detect the live/dead status of bacteria. The death of *E. coli* results in an enhancement of the membrane permeability and leakage of intracellular components outside the cells which eventually leads to a drop in conductivity and permittivity.^71^ Developments in bioimpedance instrumentation, which offer less challenging spectroscopy over higher frequencies, has resulted in recent reports that expand the frequency range from β to δ and has studied intracellular events using bioimpedance both experimentally and theoretically.^72,73^

Bioimpedance is not limited to whole body, tissues, and organs, but may also be applied to protein solutions and cell suspensions. There is a great demand for continuous, online, noninvasive monitoring modalities in bioprocessing, e.g., measuring the antibody titer in a bioreactor, for which bioimpedance possesses required criteria. The relaxation frequency of proteins is known to occur below 10 MHz, but smaller segments of protein molecules possess some degree of freedom to rotate freely and independently of the large protein molecule at higher frequencies, contributing to the total polarization of the protein. The relaxation frequency of the structural unit of protein, amino acids, and peptide is in the range of 400–3000 MHz, confirming the earlier suggestion of faster rotation of smaller segments of protein when having sufficient freedom. This results in a broad spectrum of relaxation times in proteins and larger charged biomolecules. The dielectric constant of proteins increases with increasing frequencies between 100 and 900 MHz in a concentration-dependent manner; as the concentration of a protein solution gets higher (>20%), the dielectric constant become less dependent on frequency^51^ while the dispersion frequency increases. Using an impedance-based biosensor, Oseev et al. identified a lower limit of detection for protein in δ-dispersion regimen compared to β regimen. Therefore, the sensor has a linear response in a narrow window around 280 ± 5 MHz.^74^ The dispersion of muscle, blood (erythrocytes) and fat occur at a frequency of about 4 MHz (with fat having the lowest dielectric constant reflective of its insulting nature). The dielectric constant of blood at frequencies >100 MHz is larger than muscle due to its higher water content. Measurement in the frequency of 100 MHz to 10 GHz can yield information about the electrical properties in internal cellular components, as bioimpedance in this range is not influenced by the plasma membrane or polar properties of water.

### 3.3 Equivalent Circuit Analysis

Equivalent circuit models are static, in silico representations of the spectral characteristics of the TUT, the analysis of which is intended to provide quantitative insight into the pathophysiology and/or composition of the TUT. Equivalent circuits must therefore be realistically constructed to adequately represent the dispersions associated with the physicochemical phenomena and dielectric attributes of the expected components of the TUT. Whether continuously monitoring single cells at a single interrogation frequency (BIA for cell cytometry), interrogating the health of a tissue before transplantation into a host patient, or characterizing cells or tissues, equivalent circuits must be appropriately designed to model the physiological and physicochemical properties of the TUT.

#### 3.3.1 Single Cells

**Figure 4:**
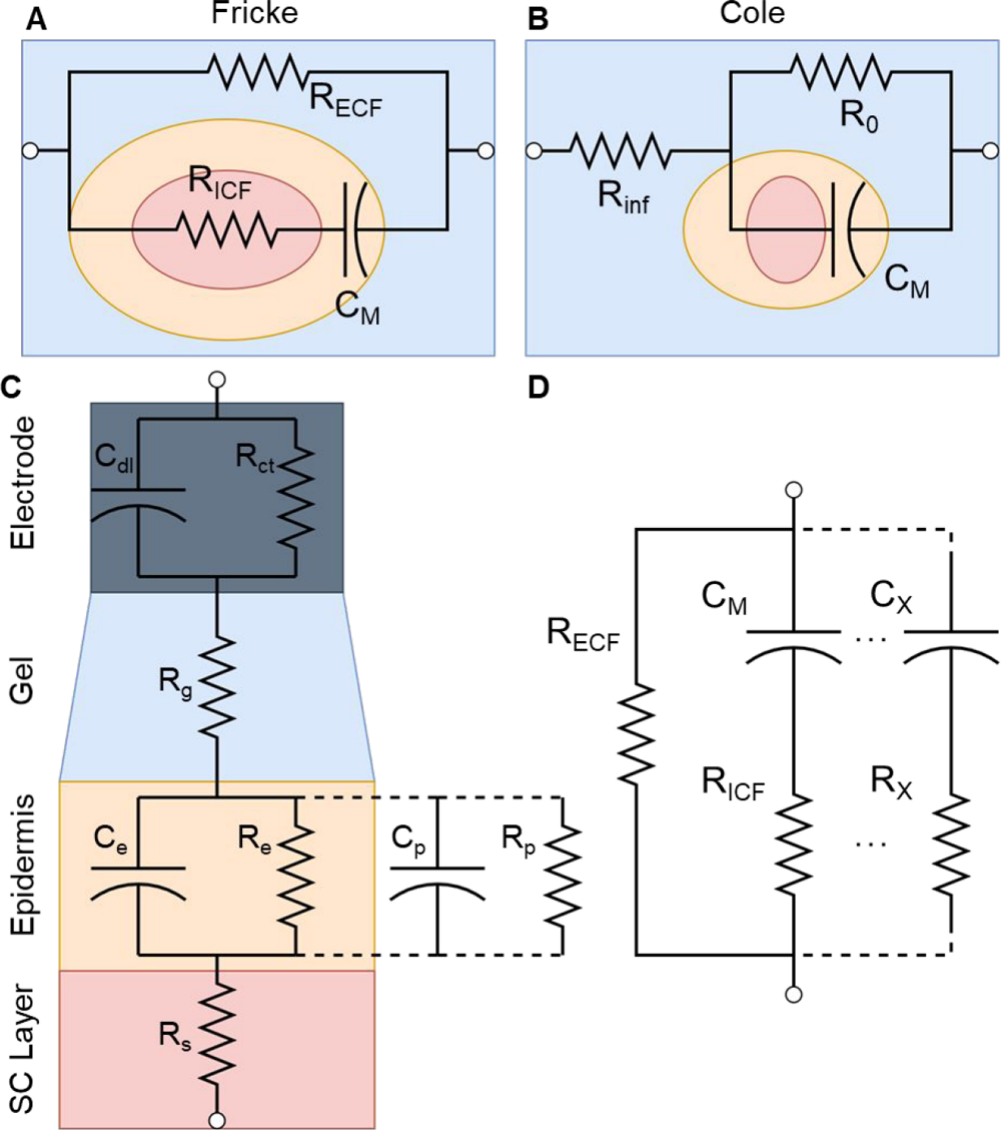
(A) Fricke and (B) Cole equivalent circuit models used for biological tissue. (C) Complex circuit model in EEG setup to model impedances from electrode, electrode/skin contact, epidermis, and subcutaneous layers. Adapted with permission under a Creative Commons CC License from ref (79). Copyright 2012 MDPI. (D) Extended Fricke model.

The generalized components of a cell that are of importance in impedimetric analysis are the cell membrane, the cytoplasm, the cytoplasmic inclusions, and the bathing media. There are two accepted basic circuits commonly used to characterize cells: the Fricke–Morse model and the Cole model. The Fricke–Morse model was one of the first equivalent circuits for biological tissue and is still commonly used today (in its purest form and in amended forms).^75^ The parameters used in this model can be directly attributed to the physiological elements of the cell (capacitive cell membrane, resistive intracellular fluid, resistive extracellular fluid) (Figure 4A). Originally published in 1925 and derived from measuring impedance of blood (8 Hz to 4.5 MHz), the Fricke–Morse model exists in several forms with varying complexity; however, the original approximation adequately models the extracellular fluid with a resistor, *R*_E_, intracellular fluid with resistor, *R*_I_, and the cell membrane with a parallel membrane capacitor, *C*_M_, and membrane resistor, *R*_M_. In the same work, Fricke proposed a model where membrane resistance was omitted and the cell membrane was modeled as purely capacitive. The impedance of such a system as a function of interrogation frequency (ω) is
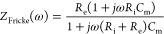
10However, in modern applications, the capacitor is replaced by a constant phase element (CPE), as the cell membrane is never a perfect capacitor given the membrane’s polarizability and pore structure that allows ions and polar molecules to flow through the semipermeable lipid bilayer.^76^ The Fricke–Morse model has found use in monitoring cell size, ionic conduction, ECW/ICW volume estimation, and cell polarization effects.

The Cole model, published slightly after inception of the Fricke–Morse model (1928) offers a more generalized biological model, where individual cellular components are not represented by passive circuit elements (Figure 4B).^77^ Given that low-frequency signals tend to traverse in the extracellular fluid and high-frequency signals tend to penetrate the cells themselves, the Cole model utilizes *R*_inf_ and *R*_0_ to signify the resistive pathways at the two frequency extremes (infinite frequency and DC). The impedance of such a system as a function of interrogation frequency is (ω)
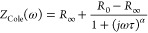
11However, in similar fashion to the Fricke–Morse model, the cell membrane is often modeled as a CPE. The Cole model is commonly utilized to measure morphological changes and heterogeneity of cell monolayers in cell-culture media.^75^ The dispersions analyzed with the Fricke–Morse and Cole models are usually within either α or β ranges, given that higher frequency measurements (hundreds of MHz to GHz) require specialized equivalent circuits.^78^ However, these simple models utilize only one circuit element with a time constant (C or CPE), therefore, to model wider frequency ranges that cover multiple dispersions or more complex samples (such as tissue), additional RC elements have been added to both the Fricke–Morse and Cole models.

#### 3.3.2 Tissues

Equivalent circuit analysis of more complex biological entities such as a single tissue layer or composite structure of multiple tissues has led to the development of multiple specialized equivalent circuits. While the Fricke–Morse or Cole models can be used to model tissue, strategies such as extension of the model via series addition of RC circuits or ladder networks have been employed, given that a tissue (in simple terms) is a connected network of cells. For example, an equivalent circuit developed to model an electrode and multiple tissue layers of skin has been rationalized via a series extension of R and RC networks by viewing the tissue as a longitudinal layered structure (Figure 4C).^79^ First, the interface of the electrode/hydrogel patch (the patch used to connect the electrode to the skin) will have a charge transfer resistance (*R*_CT_) and double-layer capacitance (*C*_DL_). The hydrogel (usually a conductive hydrogel) used to attach the electrode (usually Ag/AgCl) to the skin will have an internal gel resistance (*R*_g_). Inside the tissue, the epidermis is modeled with a resistance (*R*_e_) and capacitance (*C*_e_). Often, a second RC network is connected as a ladder to model pore capacitance (*C*_p_) and pore resistance (*R*_p_). The resistance is often a byproduct of ionic transport via sweat glands and hair follicles, while capacitance arises from lipid bilayers. The subcutaneous layer is modeled as a pure resistance (*R*_s_).^80^ The approach to developing this equivalent circuit can be used in other applications, such as measuring impedance of explanted tissues; however, no such research has been published.

**Figure 5:**
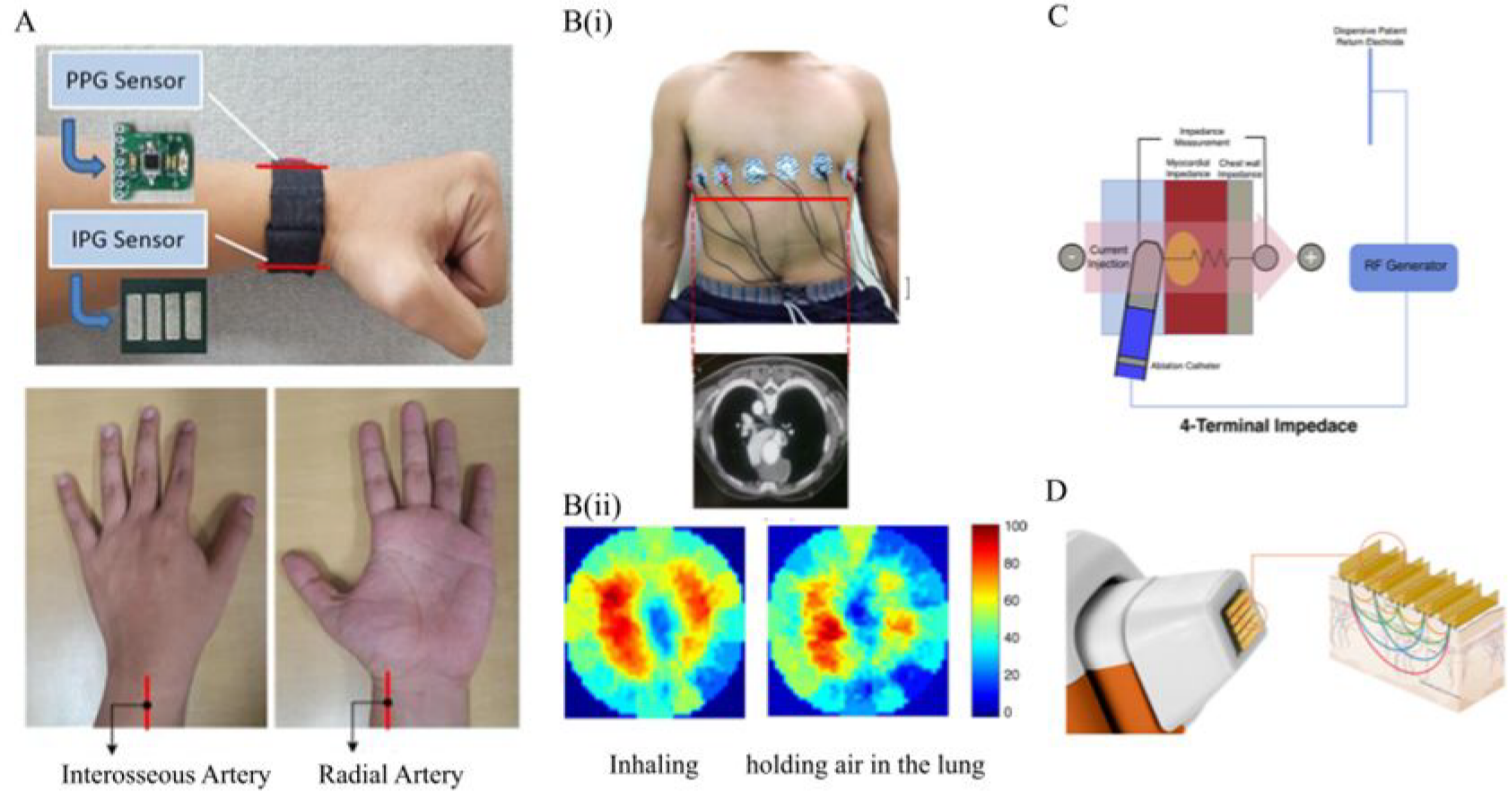
(A) Impedance plethysmography (IPG) based measurements for developing a cuffless blood pressure recorder. The band uses two sensors for IPG and PPG, collecting proximal and distal waveforms from interosseous and atrial arteries. Reprinted with permission under a Creative Commons CC License from ref (98). Copyright 2019 Springer Nature. (B) Cross-sectional image constructed using bioimpedance tomography. (i) Placement of electrodes around the chest. Reconstructed image when the case is (ii) inhaling and (iii) holding air into lungs. Reprinted with permission from ref (111). Copyright 2016 Elsevier. (C) Ablation catheter with two pairs of electrodes providing independent driving and sensing electrodes for a four-terminal impedance measurement with the purpose of determining the size of ablation. Reprinted with permission from ref (123). Copyright 2021 Elsevier. (D) Nevisense probe for measuring impedance on skin to detect melanoma.

In addition, ladder circuits have been proposed to model complex tissue—while the approach is not used to model specific regions, as in Figure 4C, these circuits have found use in full body bioimpedance measurements for measuring water content.^81^ The extended Fricke model (Figure 4D) utilizes several series RC lines in parallel to model tissues. This approach is often found in bioimpedance measurements that cover wide frequency ranges, as the model can be fitted with as many RC components (time constants) as needed. For example, a measurement made from 1 Hz to 10 MHz may utilize at least two RC ladders to account for dispersions in both the α and β frequency ranges. Recent work has demonstrated (as expected) that increasing the number of RC ladders in the circuit will lead to a better fit, however, care must be taken to not “overfit” an equivalent circuit model by addition of superfluous components.^82^ A summary of recent work utilizing bioimpedance sensors/monitoring along with the circuit model used is shown in Table 3.

**Table 3:** Use of Various Equivalent Circuits to Measure Biospecific Bioimpedance and the Frequency (and Dispersion) Ranges within Which They Were Acquired

measured parameters	frequency range	dispersion range	circuit model	AC signal amplitude	source
human body electrical shock	4 Hz to 5 kHz	α	Cole	n/a	(83)
cancerous cell discrimination	1 Hz to 1 MHz	α, β	n/a	100 mV	(84)
fibrosis	5 Hz to 100 kHz	α, β	Cole	n/a	(85)
myoblast growth/differentiation	10 Hz to 1 MHz	α, β	modified Cole	50–100 mV_p-p_	(86)
myocardial infarction/scar formation	200 Hz to 200 kHz	α, β	RC	0.198–1.98 V_p-p_	(87)
myoblast growth differentiation	500 Hz to 60 kHz	α, β	Frick, Cole	400 mV_p-p_	(75)
edema	1–500 kHz	α, β	Modified Cole	n/a	(88)
cell proliferation	3–30 kHz	α, β	Cole	200 mV_p-p_	(89)
seizure-like electrical activity	4.7 kHz to 2.0 MHz	β	Fricke, Cole	n/a	(90)
edema	20 kHz	β	Cole	n/a	(91)
identification of pulmonary nodules	50 kHz to 5 MHz	β	n/a	n/a	(92)
cell counting flow cytometry	100 kHz to 1 MHz	β	modified Fricke	250 mV	(93)
breathing activity	2 MHz to 6 GHz	β, δ	n/a	n/a	(94)
breast cancer monitoring	0.2–13.6 GHz	δ, γ	Cole	n/a	(46)
tissue characterization	0.5–26.5 GHz	δ, γ	Cole	n/a	(95)
breast cancer monitoring	0.5–50 GHz	δ, γ	Cole	n/a	(96)
cell counting flow cytometry	6.5–30 GHz	δ, γ	modified Fricke	1 V	(78)

## 4 Clinical Applications

The emergent need for more sensitive, noninvasive, portable, and cost-effective diagnostic techniques in today’s medicine has heralded use of BIA/BIS as a powerful tool for health monitoring diagnostics and prognostics. Active research, development, and clinical trials has resulted in an increasing number of clinically approved devices. Some of the most common clinical applications of BIA/BIS are introduced and summarized following.

### 4.1 Impedance Plethysmography

Impedance plethysmography (IPG) is a diagnostic tool that measures changes in the volume of an organ or tissue from recorded bioimpedance changes using skin-contact electrodes. Early in its development, IPG was used to monitor symptomatic deep vein thrombosis (DVT) in the thigh. The technique has since been expanded to monitor venous thrombosis, and in general, for diagnosis of peripheral disease of the vascular system. Despite a strong body of evidence showing the value of IPG in DVT settings, the development of compression ultrasound imaging and the emergence of D-dimer blood testing have generally overtaken IPG as the primary diagnostic method for patients suspected of having DVT.^97^ However, the simplicity and noninvasive nature of this impedance-based method has opened the way for this technique to be applied in other areas such as blood pressure measurement, wherein volumetric blood-flow data are collected from the forearm or wrist, potentially replacing the familiar blood-pressure cuff. Continuous blood pressure monitoring was achieved in the system developed by Rachin et al. by measuring the photoplethysmography and impedance plethysmography signals simultaneously from the wrist (Figure 5A). Development of such systems enable addition of a valuable health indicator to growing wearable devices such as smart watches.^98^

Impedance cardiography (ICG) (Figure 5A) is a subsidiary of impedance plethysmography utilized for the noninvasive clinical monitoring of cardiovascular parameters such as ventricular stroke volume.^99^ Ventricular stroke volume refers to the amount of blood that leaves the left ventricle per beat and is most clinically significant in two disease states: hypovolemia and heart failure. Other methods of assessing cardiac output and fluid volume status have significant limitations. The most traditional method of pulmonary artery catheterization is highly invasive and is not suitable for every situation, depending on the patient’s consciousness or intubation status and the physician’s technical skill with pulmonary artery catheterization. Another common method for the rapid assessment of fluid volume status is through physical assessment of the patient’s heart rate, orthostatic blood pressure, and urine output, among other indicators. The difficulty with this indirect assessment method is in the body’s compensatory abilities in response to its hypovolemic state. In critical care or emergency department settings, ICG is particularly useful for direct, accurate, and noninvasive clinical assessment of a hypovolemic patient’s volume status and hemodynamic stability.^100^ Allowing the measurement of beat-to-beat changes in the cardiac stroke volume from only two pairs of electrodes offers true noninvasive, real-time monitoring, which is deemed critical for the patient’s health.^101^ Thoracic impedance plethysmography (TIP), or measurement of thoracic volume for monitoring the respiratory system, has likewise developed based on IPG and has been used clinically in diagnosis of respiratory disease such apnea disorders,^102^ fluid accumulation in the lung, and cardio-respiratory complications. Fluid accumulation following heart failure is another life-threatening complication. Early detection of pulmonary (left-sided heart failure) and peripheral edema of the legs, feet, and abdomen (right-sided heart failure) through the real-time bioimpedance spectroscopy among heart failure patients can tremendously impact outcome.^103^ Additionally, localized bioimpedance analysis can yield information for clinicians in treating soft tissue injuries in the lower limbs.^104^ Body composition analyzers are another class of IPG-based devices producing key clinical information. Body composition bioimpedance analysis results in two main parameters: total tissue fluid content and cell mass. Total tissue fluid volume is a clinically important parameter for the monitoring of edema in many contexts, including overall fluid load in the case of chronic kidney disease and in peripheral edema. Tissue fluid content is an important clinical marker by which clinicians can evaluate edema, particularly longitudinally.^105^ Lymphedema, which is caused by accumulation of extracellular fluid due to dysfunction of the lymphatic system is another example where resultant body swelling can be detected with the help of bioimpedance. Since this is a common complication among patients undergoing chemotherapy, early detection for timely treatment has become critical.^106^

Instrumentation to support impedance plethysmography commonly employs one of three competing transduction technologies, strain gauge plethysmography (SGP) which measures the resistance of rubber tubes filled with displaceable mercury, photoplethysmography (PPG) which employs photo detectors, and air plethysmography (APG) which uses an air-filled cuff. Each has its own merits; however, clinical comparative tests have shown that impedance plethysmography yields similar or superior results. In a comparative study between impedance plethysmography and strain gauge plethysmography with parallel measurements taken on 145 patients (91 normal and 38 with deep venous thrombosis), IPG resulted in 4 false positives while SGP resulted in 1 false positive.^107^ In a recent study comparing the use of IPG to PPG for blood pressure detection, an IPG sensor was placed radially on the wrist, and a PPG sensor was placed on the index finger for simultaneous measurements.^108^ In comparison with traditional optical pulse transit time (PTT) techniques, the IPG sensor achieved a lower root-mean-square error (RMSE) of 8.47 ± 0.91, which was 68% lower than the model in comparison. A summary review and comparison of the competing technologies are shown below in Table 4.

**Table 4:** Comparison of Relevant Technologies Competing with IPG

	description	advantages	limitations	sensitivity/specificity for DVT
IPG	circumferential electrodes are placed on the limb; high-frequency/low voltage current is passed to measure impedance	•requires only slight skin contact	•false positive DVT analysis may result from skin/electrode contacts if scar tissue is present	75%/90%^109^
		•can be produced from soft/flexible material		
		•insensitive to location of measurement	•low sensitivity	
		•ease of use		
SGP	flexible strain gauge imbibed with mercury is placed around the limb; changes in the blood volume result in changes in the impedance of the strain gauge	•relatively inexpensive	•sensitive to temperature variations	83%/81%^110^
		•does not require skilled technician	•requires use of mercury (toxic)	
PPG	blood volume is estimated by utilizing light from LEDs and photodetectors	•high sensitivity for arterial disease	•must fit tightly onto body	94%/73.1%^109^
		•low-cost technology	•LED/photodetector consumes high power	
		•ease of use, widely used	•low sensitivity/specificity for venous reflux	
APG	chamber filled with air is placed around the limb; displacement of air is used to measure changes in blood volume	•relative ease of use	•low sensitivity for venous reflux	85%/91%^109^
		•more reproducible than SGP		
		•has replaced SGP and PPG in diagnosis of CVI		

### 4.2 Bioimpedance Tomography

Bioimpedance tomography, illustrated in Figure 5B(i), is a bioimpedance-based, clinically directed, monitoring technique often used for informing the status of bedside ventilator therapy. BIT enables medical practitioners to noninvasively measure changes in regional ventilation in the patient’s chest.^111^ The noninvasive nature and the absence of radiation exposure makes BIT particularly attractive for use in mechanically ventilated pediatric and neonatal patients. BIT may use a single (BIA) or multiple (BIS) frequencies and employs a multiplexed array of paired driving electrodes (current injection) and sensing electrodes (voltage reading) judiciously placed around the target area. Impedimetric or spectral data is analyzed, reconstructed by a computer, and presented as a heat map or other visualization format to reflect an image of the target area as shown in Figure 5B(ii). There are multiple approaches for setting and shifting driving/sensing electrodes which have been reviewed in detail.^112^ Data acquisition, number of electrodes,^113^ electrode placement, separation distance from each other, electrode size and material-tissue interface, interrogation (single or multifrequency measurements),^114^ current density, data analysis, and image reconstitution protocols are parameters which are to be optimized to acquire image with satisfying level of details.

While the early development of BIT was intended to monitor ventilation, its application has since expanded for imaging, monitoring, and assessing areas other than lung, namely brain,^114^ breast, heart, etc. Not posing the risk of X-rays, BIT offers a simple yet powerful imaging alternative. During the 2020–21 pandemic caused by SARS-CoV-2 coronavirus (COVID-19), which manifest impact on the lungs and with severe cases requiring continual mechanical ventilation, several studies explored application of BIT for monitoring lung capacity and ventilation efficiency, including using BIT for bedside monitoring of ventilation to personalize titration of positive end-expiratory pressure (PEEP) for a more effective treatment.^115^

### 4.3 Catheter-Based Bioimpedance Monitoring

Endocardial radiofrequency catheter ablation (RFCA) is a safe and effective method of treatment for cardiac tachyarrhythmias, such as atrial fibrillation, atrial flutter, and other supraventricular tachycardias.^116^ During this procedure, a destructive thermal power of maximum 50 W is delivered to abnormal cardiac tissue via electrodes on the ablation catheter to correct the myocardium function. Introduced in the 1980s with a DC current, the modern approach uses low-voltage/high-frequency (commonly 50–500 kHz) AC signals. An important metric for the success of an RF cardiac ablation is the size and depth of the lesion created in the tissue, which is affected by tissue temperature achieved, force of contact, and power delivered.^116,117^ To assess the catheter position and lesion quality, bioimpedance changes of the tissue is simultaneously collected and analyzed using the same electrodes which deliver thermal power.^117,118^

Bioimpedance as measured at the catheter tip can also be a useful parameter when assessing the quality of contact with endocardial tissue. Increases in bioimpedance can inform physicians not only of the catheter’s binary contact status but also serve as an indication of the catheter’s depth within the myocardium.^119^ Additionally, the bioimpedance data is used to render a 3D map of the cardiac structure, traditionally acquired by ultrasound, yielding a more informed treatment decision.^120,121^ Some studies report bioimpedance to be a poor predictor of contact force.^122^ To address such shortcoming, leveraging the bioimpedance catheter data with additional electrodes placed on the skin has been explored for a more accurate characterization of the lesion (Figure 5C).^123^ Moreover, a map of electrical activity of the heart can also be derived which is important for both physicians and electrophysiologist.

### 4.4 BIS for Malignancy

The biochemical and biophysical changes undergone by cells and tissues that serve to define cancer also alter their bioelectrical properties allowing such cells and tissues to be differentiated from normal tissue via their bioimpedance. Such transformation includes a wide range of changes in metabolic and mitogenic activities of cells accompanied by changes in the permeability of cell membranes, remodeling of the extracellular matrix, and changes in cell–cell and cell-matrix interactions. These changes manifest differences in the electrical properties of circulating cancer cells^124^^125^ as well as solid tumors.^126^ Use of impedance spectroscopy data adjunct to colonoscopy has improved the sensitivity and specificity of cervical cancer diagnosis.^127^ Detection of melanoma using a BIS-based system was achieved with a sensitivity rate of 96.6% in 256 out of 265 in a clinical study conducted across 22 institutes (Figure 5D).^128^

Using six discrete frequencies between 20 Hz and 5 MHz, Sun et al. measured a lower impedance in cancerous tongue tissue compared to native tongue detected only at lower frequencies (20 Hz and 50 kHz).^129^ Cancer affects the packing state of tongue cells resulting in widening of extracellular space leading to a more conductive path for ion migration at lower frequencies (reducing impedance compared to healthy parts of the same tongue as well as tongues of healthy patients).^129^ Some researchers have identified a frequency-dependent parameter, the Cole relaxation frequency (CRF), for classifying tissues and distinguishing cancerous tissue from healthy breast tissues.^130^ Defined as the frequency where the imaginary component of impedance vs frequency peaks in a *Z*_IM_–frequency plane of a Cole–Cole plot the CRF differs for cancerous vs noncancerous tissues. Based on the hypothesis that cancer decreases the polarizability of cells and hence lowers the capacitance of cells, the CRF of normal tissue (0.001–0.1 MHz) shifts to higher frequencies (0.1–2 MHz) for cancerous cells.^54^ The ability of bioimpedance measured over 1 kHz−3 MHz in differentiating between normal and different grades of cancerous cell in vivo has likewise been reported.^131,132^ The use of electric fields in this range of frequencies (100–500 kHz) to serve as treatment of malignancies (not reviewed here)^133^ should not be overlooked as the simultaneous or concomitant electrical stimulation with BIA/BIS measurement and monitoring is emerging as a powerful investigative and theranostic tool.

In a study of real-time label-free detection/discrimination of brain and tumor tissues in in vivo rat models, Jahnke et al. employed flexible microelectrode arrays to measure impedance of tissue during surgery to differentiate between healthy brain tissue and tumors.^134^ The study found that discrimination of functional brain tissue could be differentiated from tumor tissue in the range of 10–20 kHz, and an additional neuron specific impedance in the range of 100–500 kHz. Specifically, at 18.8 ± 0.5 kHz, healthy brain tissue had a characteristic impedance of 170.9 ± 8.2% higher than that of tumor tissue. In addition, healthy brain tissue exhibited an impedance plateau at frequencies above 100 kHz that was not evident in tumor tissue. Equivalent circuit analysis utilizing a complex modified Cole circuit found that all electrode related resistances and capacitances were statistically agnostic of the tissue type tested, while tissue specific parameters (*R*_Tissue_, *R*_Extra_, and *C*_Tissue_) were fitted with statistically different values between the two tissues. The researchers also discussed the importance technique when creating contact between electrodes and tissue–minor changes in the method used to contact the tissue and electrodes can create differences in measured relative impedance of 20–50%. However, if contact with the entire tissue is lost, a completely resistive relative impedance spectra is quickly measured, which can indicate to the technician that adjustments were required for effective electrode–tissue contact.

Recently, Oh et al. conducted a study to examine the ability of a bioimpedance multielectrode probe to discriminate between tissue samples with cervical intraepithelial neoplasia (*n* = 69) and those without (*n* = 54) using impedance spectroscopy over the range of 0.625–100 kHz.^135^ Using a 17-membered array of spring-loaded, gold plated electrodes and by testing each tissue sample from patients three times, statistically significant differences in reconstructed resistivity values were found in the studied frequencies ranging from 0.625 to 50 kHz. The lower resistivity demarcated by the tumor cells was posited because of destruction of tissue structure by loss of the layer of flattened cells on the surface of the sample. From these results, the researchers concluded that with a sensitivity of 94.3% and specificity of 84%, the proposed method could be a useful tool for screening for CIN. Table 5 lists a few select examples of BIA/BIS-based systems being used in clinical practice for detection of edema.

**Table 5:** Examples of FDA-Approved Medical Devices Functioning Based on Bioimpedance

bioimpedance measurement mode	clinical application	device name	FDA approval year	specification
impedance plethysmography	arterial and venous vascular diagnosis	VasoScreen 5000 by Sonicaid Inc.	1985	
	cardiac hemodynamic monitoring for the management of heart failure	BioZ thoracic impedance plethysmograph by SonoSite^136^	1997	current: 1.5 mA_eff_
				frequency: 85 kHz
	noninvasive thoracic impedance plethysmography	IQ system cardiac output monitor by Renaissance Technology^136,137^	1998	current: 4 mA
				frequency: 100 kHz
	fluid status monitoring	ZOE by NonInvasive Medical Technologies LLC^138^	2004	detect changes < 2 Ω
	noninvasive hemodynamic monitoring	Cheetah NICOM system by Cheetah Medical Inc.	2008	
	noninvasive measurement of cardiac output and its derivative	NICaS by NI-Medical^139^	2004	current: 1.4 mA
				frequency: 32 kHz
	lymphedema and fluid management: to detect edema resulting from extracellular fluid complications	L-Dex U400 and SOZO by ImpediMed^106,140^	2007 and 2018	frequencies: 3–1000 kHz (256 data points)
	monitoring distribution and changes of cerebral fluids for identification of brain pathologies	Visor by Cerebrotech Medical Systems	2018	frequencies: 30–310 MHz
electrical impedance tomography	mammography	T-Scan 2000 by TransScan Medical^141^	2000	voltage: 1–2.5 V
				frequencies: 0.1–100 kHz
	provides information on the regional distribution of ventilation	ENLIGHT 1810 by Timpel S/A^142^	2018	
catheter-based bioimpedance monitoring	creation of 3-D cardiac models of the heart’s electrical activity	FlexAbility ablation catheter, sensor enabled by Abbott^143^	2017	
	monitoring the effect of energy delivered during cardiac ablation procedures	DirectSense technology by Boston Scientific^144^	2020	
bioimpedance spectroscopy	early detection of melanoma	Nevisense by SciBase Nevisense system^128^	2017	frequencies: 0.001–2.5 MHz

## 5 BIS and Telehealth

### 5.1 Wearable Biomedical Devices

In recent years, miniaturization of transistor size in accord with Moore’s Law has yielded the ability to fit greater capabilities onto small wearable devices, resulting in wearable biosensors and the implementation of novel tele-medicine systems. The introduction of optical technologies into smart watches to measure heart rate, oxygen tension, and even ECGs, has led to new avenues for personalized medicine. Medical care providers are now equipped with a set of tools that allow patients to passively and continuously collect large amounts of data from biomedical sensors outside of the medical care setting as opposed to collecting singular pieces of data at each appointment. Development of various wearable/miniaturized biomedical sensors in the forms of smart watches, smart bands, smart scales, smart clothing attachments, has yielded several FDA approved devices capable of measuring biometrics including heart rate, heart rate variability, respiration rate, chest fluids, body fat, body water %, muscle mass, and bone mass. A summary of novel wearables and their applications is shown in Table 6.

**Table 6:** Summary of Available Wearable Biosensors^a^

**Sensor Name**	**Company**	**Application**	**Hardware**	**Sensors**	**Computed Biometrics**	**Comms.**	**Footprint (mm)**	**Batt. Life**	**Applied Signal**	**FDA Approved**
BX100	Philips	COVID-19 monitoring	Chest worn patch	2 ECG BIA electrodes	Activity level/type, posture	BT	96 × 61	5 days	16 μA	Y
									40 kHz	
Body+	WiThings	Smart scale	Stand on scale	4 BIA electrodes	BF, BMI, Water %, MM, BM	BT/Wifi	327 × 327 × 23	18 months	0.5 mA	N
									50 kHz	
Cova2	toSense	Clinical & remote monitoring	Necklace	2 ECG/BIA electrodes	SV, CO, CF, ECG, HR, RR	BT/Wifi	n/a	n/a	4 mA	Y
									70–100 kHz	
Aura Band	Aura	Sports monitoring	Wrist Band	2 BIA electrodes	BC, hydration	BT	44 × 25 × 5	7 days	n/a	N
									10–500kHz	
Ebio Unit	Shimmer	COVID-19 and sports monitoring	Chest worn device	5 ECG/BIA electrodes	RR, RV, CF	n/a	n/a	48 h	n/a	N
									32 kHz	
Band2	Inbody	Sports monitoring	Wrist band	2 BIA electrodes	BF, MM	BT	19 × 26 × 10	14 days	n/a	Y
Enduro	Physioflow	Cardiac output monitoring	Chest worn device	6 BIA electrodes	CO	BT	115 × 85 × 18	6 h	4.5 mA	Y
									66 kHz	
μCor3	Zoll Medical	Cardiac event monitoring	Under arm or chest patch	2 ECG/BIA electrodes	TI, HR, RR	BT	69 × 53 × 12	5 days	n/a	Y
									0.5 kHz-2.5 GHz	
ROBIN	Onera Health	Sleep apnea detection	Chest work patch	4 BIA electrodes	RR	BT	65 × 20 × 130	n/a	0.1 mA	N
									8–160 kHz	
Vantage M	Polar	Sports monitoring	Wrist band	4 BIA electrodes	HR	BT	46 × 46 × 12.5	1.25 days	n/a	N
ONE SMARTDIET	ONESOFTDIGM	Body composition analysis	Hand-held	4 BIA electrodes	BF, MM, BMI, BWV	BT/Wifi	75 × 31 × 17	7 days	150 μA	N
									50 kHz	
Galaxy Watch 4	Samsung Electronics	Body composition analysis	Wrist band	2 BIA electrodes	BF, MM, BWV	BT	n/a	n/a	30 μA	N
									50 kHz	

a Abbreviations: BIA, Bioimpedance analysis, BC: total body composition, BF: body fat, BM: bone mass, BT: Bluetooth, BWV: body water volume, CF: chest fluids, CO: cardiac output, HR: heart rate, LBM: lean body mass, MM: muscle mass, RR: respiratory rate, RV: respiration volume, SV: stroke volume, TI: thoracic impedance).

From the table, most devices occupy a small footprint - a necessity if a device is to be comfortably “wearable”. Additionally, available documentation of these devices indicates that all bioimpedance measurements are taken in the α and/or β frequency windows because (i) the instrumentation required for higher frequency measurements is expensive and often too bulky, and (ii) these frequency ranges can give excellent insight into health monitoring applications.

Released in 2020 with a 510(k) FDA clearance, the Philips BX100 (Philips N.V., Amsterdam, Netherlands) sensor system was designed to be implemented in hospital settings with large amounts of COVID-19 positive patients. The chest worn patch, which houses two ECG bioimpedance electrodes, measures vital signs including respiration and heart rate, posture, activity level, and ambulation. The device itself is manufactured from disposable foam and requires a single CR2032 3 V coin cell battery (capable of operating continuously for 120 h). The waterproof device utilizes a small electrical current (16 μA, 40 kHz) to detect heart rate within 10% and respiratory rate within ±3 rpm.

Released in 2017 with 510(k) FDA clearance, the toSense Cova2 (toSense, San Diego CA, USA) is a novel wearable device made in the form factor of a necklace designed to be used in remote patient monitoring, postdischarge monitoring, and clinical monitoring applications. Two disposable electrodes on the back side of the necklace are attached to the patient’s upper chest, where ECG and impedance waves are measured to elicit information about the heart rate, respiration rate, skin temperature, stroke volume, posture, and cardiac output. A recent study of in-home monitoring of vital signs in the intended use population of the device indicated that 73% of participants felt better about their health and had a better picture of their health as a result of using the device.^145^

In the area of athletics, several novel wearable devices have been developed to track athletic performance. The Aura Band (Aura Devices, Wilmington DE, USA) is sold as an advanced fitness tracker that integrates several sensors (optical IR, accelerometer, bioimpedance electrodes) to present a full body composition analysis (fat, muscle mass, minerals, and hydration). The system uses a wide range of frequencies (10–500 kHz) to capture data in both the α and β frequency windows. The system uses the simplified Fricke model (section 3.3) to extract and report intracellular and intravascular water.

Initial studies by researchers at Samsung led to the development of a smartphone connected (Bluetooth) hand-held bioimpedance device capable of discerning body fat and skeletal muscle by using a 4-electrode system at frequencies ranging from 5 to 200 kHz.^146^ In a 568 subject study, the calculated skeletal muscle showed high correlation with dual-energy X-ray absorptiometry reference measurements at the 3 tested locations: palm-to-palm (0.96), finger-to-finger (0.95), and palm-to-knee (0.94). Further development into a deployable wearable utilized the Samsung Electronics BioProcessor2 (a system developed to calculate body fat, lean body mass, and body water via 2- and 4-point bioimpedance measurements) in a clinical study to evaluate accuracy of the chip when deployed in a wrist worn device.^147^ The system, which utilizes a 30 μA/50 kHz interrogation signal is currently under development at Samsung. In a clinical study with 203 patients, the implementation of the BioProcessor2 yielded results closely correlated to a reference bioimpedance analyzer (DEXA, GE Lunar Prodigy) with an *R*^2^ of 0.8085.

Bioimpedance can also be used as a biometric, similar to a fingerprint or retinal scan. With a focus on the ability to correctly detect patient identity to avoid incorrect surgical procedure, a recent study characterized a device able to recognize the user based on bioimpedance measurements taken from the wrist.^148^ Bioimpedance measurements taken at 10 and 100 kHz resulted in a system capable of accuracies above 90% in a study population of 100 patients. While the feasibility of utilizing bioimpedance over long periods of time for verifying identity may be farfetched (due to tissue aging, disease, weight gain/loss, hydration, etc.), the concept is still quite remarkable.

While the miniaturization of chip sizes has burgeoned a new age of bioimpedance-monitoring wearable devices, these results are not sufficient for a self-diagnosis or prescription. A professional clinician is still required. For this reason, the implementation of the Internet of Medical Things has similarly burgeoned, making data available to physicians in higher volume and accuracy.

### 5.2 Internet of Medical Things (IoMT)

**Figure 6:**
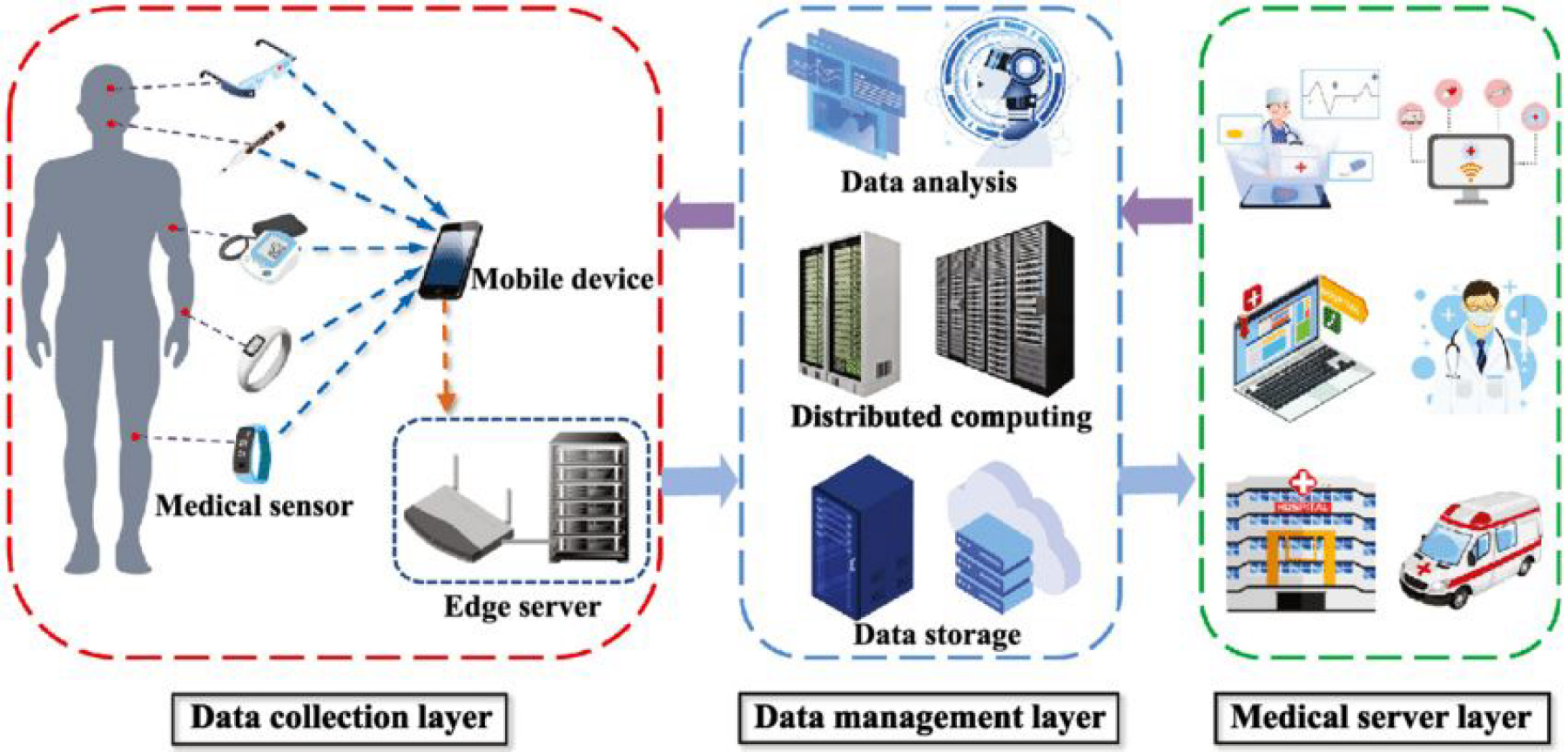
Three-layer architecture used in IoMT. Reprinted with permission from ref (149). Copyright 2020 Elsevier.

The Internet of Things (IoT) serves to connect sensors and input–output devices via the Internet to allow for remote monitoring and control. The integration of medical equipment, wireless sensors, Internet, and hospitals with IoT technology/protocols, known as IoMT presents a new paradigm is healthcare, broadening telehealth. The IoMT boom is being driven by smart wearable devices, home-use medical devices, and point-of-care kits, several of which use bioimpedance. However, given that medical record laws are governed by the US HIPAA Privacy Rule, IoMT systems are subject to more scrutinous data transmission/storage security. Usually, IoMT systems can be modeled in three- or five-layer architecture (with the inclusion of adaption and transport layers) (Figure 6): perception layer, where sensor measurements are made and from which data are transmitted; network layer that connects the transmitted sensor data to other network devices using routers via Internet Protocol (IP); application layer, where patient-specific and/or physician-specific interfaces enable interactivity.

This architecture establishes a direct line of data transmission between patient and healthcare provider, which is fraught with security concerns. Biohacking that seeks to disrupt normal device function can potentially result in patient death. For example, Bluetooth hackers can achieve denial-of-service (DoS),^150^ WiFi eavesdropping can compromise data integrity,^151^ and the absence of encryption protocols can compromise sed/receive messages.^152^ The challenge of managing large data volume, privacy policies, and sensor interoperability may be addressed by semantic medical IoT (SM-IoT).^153^ The CryptoCurrency Security Standard (CCSS) is now being adopted and adapted by some in the IoMT device space.^154^

The Philips BX100 biosensor (previous section), designed for implementation in hospital settings, uses BLE 4.2 and WiFi to wirelessly transmit recorded data at 1 min intervals to a router running Philips’ IntelliVue Guardian Software. Each router (data management layer), which can connect to 16 biosensors simultaneously will transmit data to the medical service layer: the software automatically receives and tracks data from the deployed sensors, identifies deviations/deterioration of vital signs, and alerts the caregivers to act. From a security standpoint, data is encrypted via 128-bit Advanced Encryption Standard (AES) for both local storage on the device and data transmitted over Bluetooth/WiFi. A recent clinical study utilizing 44 adult patients in the emergency department observation unit (EDOU) of Brigham and Women’s Hospital demonstrated the feasibility of implementing such systems in a hospital setting as well as the implications of continuously monitoring large numbers of patients (particularly during a mass triage situation such as COVID-19).^155^

An IoMT-enabled, standalone bioimpedance analyzer that uses eight electrodes placed around the abdomen and upper chest, can operate at an extremely wide bandwidth (100 mHz to 10 MHz) using a miniaturized instrumentation (not entirely wearable) footprint. A web-based user interface allows for real time data visualization of several devices via a wireless or local area network. The embedded software, developed in C language, provides large data management features, and the web server runs on the open-source NGINX engine. Web clients can access the data through Ethernet, WiFi or USB port (for system maintenance). They are able to set different configuration parameters for the bioimpedance measurements such as mode (impedance excitation stepped-sine or multisine signal), frequency range, amplitude, etc.^156^

Zhang et al. has presented a remote health monitoring system that integrates and tracks temperature, respiration, blood oxygen saturation, pulse rate, blood pressure, and ECG data. Bioimpedance was used to measure respiration via the thoracic impedance method, which applies a current signal of 10k-100 kHz between two electrodes placed on the chest. The sensors were connected via BLE to a monitoring center which in turn was connected to the Global System for Mobile Communications (GSM) for real-time data tracking. Tested on six volunteers, the system proved reliable; nonetheless, further research regarding large data management and analysis speed were areas for further development.^157^

## 6 Future of BIA/BIS for Human Health Applications

Given the shift in healthcare archetype (particularly in the 21st century), where technological advancements have pushed the field toward personalized approaches, bioimpedance spectroscopy as a clinical monitoring tool has a bright future. Simple wearable bioimpedance devices and the development of the IoMT reject the need for the patient to be in the clinic for testing; data can be gathered continuously over time as the patient carries on normal activities. This favors diagnoses based on pathophysiologic trending data over STAT or urgently generated data. Although BIA/BIS has not been widely adapted in many traditional healthcare monitoring systems, the miniaturization of devices and ability to capture multiple types of data on a single device presents opportunity for integration into more clinical platforms.

Reduction in the size and configuration of interrogating electrodes^147,158^ and advances in instrumentation^159,160^ promise improved spatial resolution. The feasibility of miniaturized excitation electronics has led to the deployment of wearable microanalytical systems. The use of multiplexed, multielectrode techniques and sophisticated imaging, such as electric field focusing, promise improved thermography.^19,161^ A recent patent described a miniaturized wearable device wherein both ECG signals and bioimpedance signals were measured using a shared pair of electrodes in contact with the skin.^162^ By using the same probing circuit and multiplexing between interrogation methods, the overall footprint of a device was miniaturized and data maximized. Such miniaturized devices can be integrated into traditional wearables such as smart watches and patches and also into clothing and jewelry.

There is growing interest in fusing multimodal data (data from multiple, often unrelated measurement modalities) via mathematical algorithms and/or the tools and techniques of artificial intelligence (AI) to yield derivative data and engage predictive analytics such as tissue or patient stratification. For example, it was reported that blood glucose concentrations can be calculated from fusing data collected from ECG and bioimpedance electrodes. Individually, merit has been shown regarding noninvasive glucose measurements using ECG (analyzing QRS complex, QT time interval, and ST segment^163^) and bioimpedance (resonant frequency method^164^), however, by fusing the data sets using machine learning algorithms to arrive at a single output can reduce deviation from artifacts (such as movement) and yield more clinically accurate results. Further incorporation of additional sensing modalities (such as optical or electrochemical) will result in the ability not only to detect more biomarkers/biosignals, but the ability to fuse all these data to create a better picture of the patient’s overall health status.^165^

Furthermore, the development of field-focusing will allow the bioimpedance properties of specific volume elements (voxels) within complex organs and/or tissue to be addressed, measured and monitored.^166^ Novel applications based on BIS are emerging in conjunction with the use of artificial intelligence, e.g., deep learning neural networks and machine learning algorithms to achieve tissue classification/stratification. BIA/BIS collected from skin has been used for gesture recognition with a broad application outlook including supporting disabled patients.^167^ BIA/BIS has been also suggested for a unique signature for identity authentication.^168^

The key advantages of BIA/BIS methods in clinical applications are the simplicity of its deployment and its noninvasiveness. Alternative tissue biomonitoring methods, often with better precision and accuracy, come with considerable technological complexity and expense. For example, CT and fMRI may produce superior images compared to BIT, but require costly equipment and maintenance, and a high level of technical training to analyze and interpret data. As discussed in sections 3.3 and 5, technological advancement in collecting and analyzing impedance data is being intensely investigated to improve both real-time data capture and interpretation enabled by AI. The relative nature of bioimpedance, specifically the need for and the differences among impedance baseline from patient-to-patient presents a hurdle to its universality. Using BIS as an adjunct to other diagnostic methods and in combination of artificial intelligence has led to the possibility of producing predictive models to enhance our prognostic capability.^169^
